# Alternating suture and staple placement for linear high-tension scalp wounds: A video walk-through

**DOI:** 10.1016/j.jdin.2024.03.027

**Published:** 2024-04-23

**Authors:** Spencer P. McClure, Edward W. Seger, Atieh Jibbe

**Affiliations:** a Division of Dermatology, University of Kansas Medical Center, Kansas City, Kansas; b Department of Dermatology, Scripps Health, San Diego, California

**Keywords:** dermatologic surgery, Mohs, reconstruction, scalp, surgery, staples

## Challenge

Skin cancer of the scalp is common, and the limited tissue laxity of this region often presents a reconstruction conundrum. Secondary intention healing is viable for smaller, shallow tumors in patients without significant hair growth, but most defects require reconstruction. For defects where a linear closure is preferred, it is important to minimize dermal and superficial tension to reduce risk of dehiscence and track marking.^1^ Epidermal staples have been advocated to reduce track marks, but placing secure dermal sutures often represents the greater challenge with these high-tension regions.^2^ We present an efficient stepwise technique for closure of linear scalp defects.

## Solution

**Fig 1:**
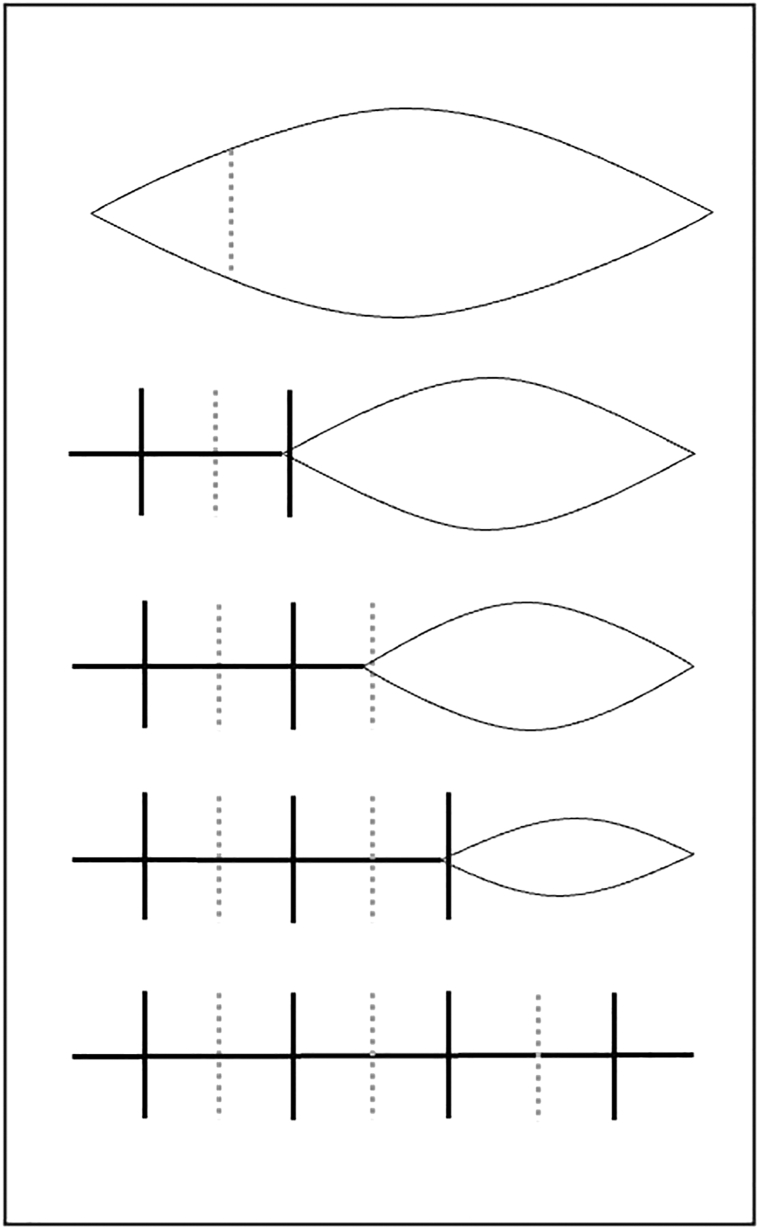
Stepwise placements of dermal sutures and epidermal staples. A linear ellipse defect with 3:1 length-to-width ratio is depicted. First, a buried horizontal dermal suture (gray dotted line) is placed near one of the apices. This suture partially closes the ellipse and reduces the width of the defect. Next, epidermal staples are placed on either side of this dermal suture. This secures the lateral apex of the closure and further reduces the width of the defect. By continuing this stepwise closure, each subsequent dermal suture is placed under less tension than it could have been if the full dermal closure was completed prior to the epidermal closure. Staples are subsequently removed at 3 weeks.

Tissue laxity is assessed, and a linear reconstruction is designed with an approximately 3:1 length-to-width ratio. The depth of the defect is extended to the subgalea to allow for undermining in an avascular plane, and hemostasis is achieved using electrocautery. A buried vertical mattress suture (such as 3-0 poliglecaprone 25) is placed near the lateral apex of the ellipse (Fig 1; Video 1, available via Mendeley at https://data.mendeley.com/datasets/3jdn6x32sg/1). Placement of the first dermal suture near the apex minimizes tension on the absorbable suture as compared with beginning at the midline (the traditional “rule of halves”). Epidermal staples are then used on either side of the dermal suture. The second buried vertical mattress is then placed, and the process is repeated until the defect is closed. Using staples during the process of dermal closure reduces tension on subsequent deep sutures by reducing defect width. Staples are removed at 3 weeks, which further reduces risk of dehiscence.

## Conflicts of interest

None disclosed.
